# Methicillin-Resistant *Staphylococcus aureus* ST9 in Pigs in Thailand

**DOI:** 10.1371/journal.pone.0031245

**Published:** 2012-02-17

**Authors:** Jesper Larsen, Maho Imanishi, Soawapak Hinjoy, Prasit Tharavichitkul, Kwanjit Duangsong, Meghan F. Davis, Kenrad E. Nelson, Anders R. Larsen, Robert L. Skov

**Affiliations:** 1 Department of Microbiological Surveillance and Research, Statens Serum Institut, Copenhagen, Denmark; 2 Department of Epidemiology, Johns Hopkins Bloomberg School of Public Health, Johns Hopkins University, Baltimore, Maryland, United States of America; 3 Bureau of Epidemiology, Department of Disease Control, Ministry of Public Health, Nonthaburi, Thailand; 4 Department of Microbiology, Faculty of Medicine, Chiang Mai University, Chiang Mai, Thailand; 5 Department of Environmental Health Sciences, Johns Hopkins Bloomberg School of Public Health, Johns Hopkins University, Baltimore, Maryland, United States of America; University of Iowa, United States of America

## Abstract

**Background:**

Methicillin-resistant *Staphylococcus aureus* (MRSA) is an important nosocomial and community-associated pathogen. Recently, livestock-associated MRSA (LA-MRSA) has emerged and disseminated in Europe and North America and now constitutes a considerable zoonotic burden in humans with risk factors of pig exposure, whereas the extent of the livestock reservoir is relatively unknown on other continents.

**Methodology/Principal Findings:**

From March through April 2011, MRSA was identified in pigs from 3 out of 30 production holdings in Chang Mai Province, Thailand. Representative isolates were subjected to molecular characterization and antimicrobial susceptibility testing; all isolates had genotypic and phenotypic characteristics of LA-MRSA previously characterized in the region: they belonged to ST9, lacked the *lukF*-*lukS* genes encoding Panton-Valentine leukocidin, and were resistant to multiple non-β-lactam antimicrobials. However, unlike other Asian LA-MRSA-ST9 variants, they were *spa* type t337 and harbored a different staphylococcal cassette chromosome *mec* IX.

**Conclusions/Significance:**

A novel MRSA-ST9 lineage has been established in the pig population of Thailand, which differs substantially from LA-MRSA lineages found in other areas of the continent. The emergence of novel LA-MRSA lineages in the animal agriculture setting is worrisome and poses a serious threat to global public health.

## Introduction

Recent reports of methicillin-resistant *Staphylococcus aureus* (MRSA) in livestock, particularly pigs, and in humans with contact to livestock provided the first evidence of the existence of a true livestock-associated MRSA (LA-MRSA) reservoir throughout Europe [1], [2]. Since then, LA-MRSA has been identified in Canada [3], United States [4], China [5]–[7], Malaysia [8], and Korea [9].

LA-MRSA isolates have unique characteristics that distinguish them from community- and healthcare-associated MRSA (CA-MRSA and HA-MRSA, respectively) isolates (reviewed in [10]): the majority of isolates lacks human niche-specific genes including toxins such as Panton-Valentine leukocidin (PVL) and other enterotoxins; they carry unique staphylococcal chromosomal cassette (SCC)*mec* elements encoding β-lactam resistance; they exhibit co-resistance to many non-β-lactam antimicrobials (e.g., antibiotics and metals) including those commonly used in animal production.

Interestingly, multi-locus sequence typing (MLST) showed that closely related sequence types (STs) within clonal complex (CC)398 predominate in Europe and North America, whereas the vast majority of LA-MRSA isolates from Asia belongs to CC9 (ST9 and single-locus variants thereof).

There is still a comparative lack of information on the emergence of LA-MRSA and its burden on human disease in South-East Asia. The purpose of this study was to investigate whether pigs constitute an MRSA reservoir in Thailand. We present evidence that a novel MRSA-ST9 lineage has been established in the pig population of Thailand, which differs substantially from LA-MRSA lineages found in other areas of the continent.

### Analysis

From March through April 2011, nasal swabs were taken from 4 pigs from each of 20 small-scale and 10 large-scale confined production holdings located in Chiang Mai Province (Table 1). The different pig production systems were categorized based on the definitions (i.e., size of herds, production goals, and husbandry management) of the Food and Agriculture Organization of the United Nations [11]. The herd sizes ranged from 28 to 120 animals (mean, 50 animals) and from 240 to 3,350 animals (mean, 861 animals) on small- and large-scale confined production holdings, respectively. This study was approved by the local animal ethics committee and the Faculty of Medicine, Chiang Mai University (protocol no. 41/2553) and was performed according to national guidelines for animal care. All 30 holdings volunteered to participate in the study. The 4 nasal swabs from each holding were pooled and incubated overnight in Mueller-Hinton broth supplemented with NaCl (6.5%) followed by overnight incubation in tryptic soy broth (TSB) supplemented with cefoxitin (3.5 ug/ml). Ten microliters of TSB broth were plated on Baird-Parker agar (Oxoid, Basingstoke, United Kingdom) and incubated for 24 h at 37°C. Presumptive MRSA colonies were subcultured onto 5% sheep blood agar and evaluated phenotypically by coagulase and catalase production and dextrose and mannitol fermentation and genotypically by 16S rRNA gene sequencing and presence of *mecA*, using PCR assays and primers that have been described elsewhere [12].

**Table 1:** Distribution of methicillin-resistant *Staphylococcus aureus* among pig production holdings in Chang Mai Province, Thailand.

	Proportion (%) of positive holdings	
Village	Small-scale confined production holdings	Large-scale confined production holdings
Rongkhud	0/1 (0)	
Sunkuangcome	0/2 (0)	
Tamao	3/15 (20)	
Tungtor	0/2 (0)	
Moo 1		0/2 (0)
Moo 2		0/1 (0)
Moo 3		0/2 (0)
Moo 4		0/1 (0)
Moo 5		0/2 (0)
Moo 6		0/1 (0)
Moo 7		0/1 (0)
Total	3/20 (15)	0/10 (0)

A single MRSA colony from each positive holding was selected for further analysis. Staphylococcal protein A *(spa)* typing was performed using the Ridom Staph Type standard protocol (http://www.ridom.com) and the Ridom SpaServer (http://spa.ridom.de/index.shtml), and MLST types were determined as described previously [13]. STs were assigned through the MLST database (http://www.mlst.net). The eBURST algorithm was used to assign individual STs to specific CCs (http://www.mlst.net). Presence of *lukF-PV* and *lukS-PV* genes (encoding PVL) was investigated by a PCR assay that has been reported elsewhere [14]. Typing for the staphylococcal cassette chromosome *mec* (SCC*mec*) was performed using a PCR-based multiplex assay described by Kondo et al. [15].

The antimicrobial susceptibility profiles (spectinomycin, gentamicin, kanamycin, tobramycin, rifampin, trimethoprim-sulfamethoxazole, clindamycin, erythromycin, linezolid, chloramphenicol, mupirocin, ciprofloxacin, minocycline, tetracycline, and fusidic acid) were determined by the agar dilution method using Neo-Sensitabs (Rosco, Taastrup, Denmark), in accordance with the Clinical Laboratory Standards Institute guidelines [16]. Screening for reduced susceptibility to glycopeptides was performed on brain-heart infusion agar supplemented with 5 ug/ml teicoplanin followed by testing of positive MRSA isolates using Etest glycopeptide-resistance detection strips (vancomycin, 32-0.5 ug/ml; teicoplanin, 32-0.5 ug/ml) (bioMérieux, Marcy l'Etoil, France) as described by Fitzgibbon et al. [17].

Three (15%) of 20 small-scale confined production holdings were positive for MRSA (Table 1); analysis of a single isolate from each of the 3 positive holdings showed that they belonged to *spa* type t337 and carried SCC*mec* IX. In comparison, none of the 10 large-scale confined production holdings were positive for MRSA (Table 1). The t337 isolates had genotypic and phenotypic characteristics of LA-MRSA: they belonged to ST9 (CC9), were resistant to 9 of 16 non-β-lactam antibiotics (gentamicin, kanamycin, tobramycin, clindamycin, erythromycin, chloramphenicol, ciprofloxacin, minocycline, and tetracycline, with the exception of 1 isolate that was susceptible to erythromycin), and lacked the *lukF*-*lukS* genes encoding PVL.

## Discussion

This study provides the first evidence of a porcine reservoir of MRSA-ST9-IX in Thailand. However, the extent and diversity of the MRSA reservoir in animals remains speculative because of the limited sample size and analysis of only a single colony per holding. It should be noted that the 3 positive holdings were located in the village of Tamao (Table 1), which supports limited geographical dispersal of this clone. It is also possible, however, that this rather specific geographic pattern is attributable to the fact that holdings from Tamao were overrepresented in this study, which may have increased the likelihood to recover MRSA.

The origin of LA-MRSA-ST9-IX (*spa* type t337) in Thailand is not known. The distribution of *spa* types associated with LA-MRSA-ST9 shows rather distinct geographic patterns with t899 and t4358 being the most common *spa* types in pigs from China and Malaysia, respectively [5]–[8]. In addition, SCC*mec* V predominates among LA-MRSA-ST9 from other Asian countries [5], [6], [8]. Conversely, the antimicrobial susceptibility profiles of LA-MRSA-ST9 isolates from different Asian countries, including Thailand, are highly homogenous, with the important exception that Neela et al. [8] observed high rates of resistance to trimethoprim-sulfamethoxazole (100%) in LA-MRSA-ST9 from Malaysia.

To date, SCC*mec* IX has only been described in a single MRSA-ST398 isolate (*spa* type t034) recovered from a Thai participant at the 19th International Pig Veterinary Conference in Denmark [18], [19], which suggests that SCC*mec* IX is geographically restricted. Notably, the 18-kb J1 region of the large SCC*mec* IX (43,675 bp) contains a *cadDX* operon, a *copB* gene, and two arsenate resistance operons, *arsRBC* and *arsDARBC* [19]. The presence of multiple genes that confer resistance to both metals and non-β-lactam antibiotics in LA-MRSA isolates may reflect antimicrobial use patterns in animal agriculture, and may affect the emergence, dissemination, and persistence of methicillin resistance in this setting through co-selective processes.

Methicillin-susceptible *S. aureus* (MSSA) lineages with *spa* types t034 (CC398), t1333 (CC30), and t337 (CC9) are predominant in the pig population [20], and it is therefore possible that the geographically confined LA-MRSA-ST9-IX variant in Thailand was derived from an LA-MSSA-ST9 strain belonging to *spa* type t337 through local acquisition of SCC*mec* IX. If this is the case, other MSSA CC9 (and CC30) populations circulating in the animal agriculture setting around the world may also have the propensity to acquire SCC*mec*, thereby expanding the porcine MRSA reservoir.

The presence of SCC*mec* IX in LA-MRSA CC9 and CC398 from Thailand suggests that SCC*mec* transfer can occur between these lineages, although the direction of transfer remains unclear. However, it is also possible that other MRSA types or methicillin-resistant coagulase-negative staphylococci are the source of SCC*mec* IX in *S. aureus* CC9 and CC398. Additional studies investigating the role and direction of horizontal transfer in the evolution of *S. aureus* CC9 and CC398 are currently under way.

The carriage rate among humans with contact to pigs was not investigated in this study. Nevertheless, zoonotic transmission of LA-MRSA-ST9 from pigs to humans with contact to pigs has been previously reported in China and Malaysia [5], [8], and it is therefore possible that the LA-MRSA-ST9-IX variant in Thailand also constitutes a zoonotic burden in humans with risk factors of pig exposure.

Cases of MRSA-CC9 carriage and infections in human populations have not been reported in Thailand, unlike in other countries of the region where MRSA-ST9 (*spa* type t899) and MRSA-ST834 have been isolated from sporadic cases of infection in China and Cambodia, respectively [21], [22]. Interestingly, MRSA-ST834 was subsequently found to be the predominant CA-MRSA type in Cambodia [23]. CA-MRSA-ST834 has unique characteristics compared with LA-MRSA-ST9: it is a double-locus variant of ST9; it harbors SCC*mec* IV; it exhibits high rates of resistance to rifampin (90%) and trimethoprim-sulfamethoxazole (90%), whereas rates of resistance to gentamicin (15%), chloramphenicol (3%), and ciprofloxacin (20%) are low [23]. These findings provide support for the view that distinct variants of MRSA-CC9 circulate in the community and animal agriculture settings as has been suggested for *S. aureus* CC398 [24].

In conclusion, our findings demonstrate that MRSA is present in the Thai pig population. However, the extent of the porcine MRSA reservoir and its implications for MRSA carriage and disease in humans in Thailand and other Asian countries has still to be determined.
